# Efficient generation of bispecific IgG antibodies by split intein mediated protein trans-splicing system

**DOI:** 10.1038/s41598-017-08641-3

**Published:** 2017-08-21

**Authors:** Lei Han, Junsheng Chen, Kai Ding, Huifang Zong, Yueqing Xie, Hua Jiang, Baohong Zhang, Huili Lu, Weihan Yin, John Gilly, Jianwei Zhu

**Affiliations:** 1 Engineering Research Center of Cell & Therapeutic Antibody, MOE, School of Pharmacy, Shanghai Jiao Tong University, Shanghai, China; 2 Jecho Laboratories, Inc, Frederick, MD USA; 3 School of Life Science, East China Normal University, Shanghai, China; 4 Jecho Biopharmaceuticals Co., Ltd, Tianjin, China

## Abstract

Many methods have been developed to produce bispecific antibodies (BsAbs) for industrial application. However, huge challenges still remain in synthesizing whole length BsAbs, including their assembly, stability, immunogenicity, and pharmacodynamics. Here we present for first time a generic technology platform of generating bispecific IgG antibodies, “Bispecific Antibody by Protein Trans-splicing (BAPTS)”. Different from published methods, we assembled two parental antibody fragments in the hinge region by the protein trans-splicing reaction of a split intein to generate BsAbs without heavy/heavy and light/heavy chain mispairing. Utilizing this simple and efficient approach, there have been several BsAbs (CD3×HER2, CD3×EGFR, EGFR×HER2) synthesized to demonstrate its broad applicability. Correctly paired mAb arms were assembled to form BsAbs that were purified through protein A affinity chromatography to demonstrate industrial applicability at large scale. Further, the products were characterized through physical-biochemistry properties and biological activities to confirm expected quality of the products from “BAPTS”. More importantly, correct pairing was confirmed by mass spectrum. Proof-of-concept studies with CD3×HER2 BsAb (T-cell recruitment) demonstrated superior bioactivity compared with trastuzumab. The results of undetectable mispairing and high biological activity have indicated that this method has the potential to be utilized to manufacture BsAbs with high efficiency at industrial scale.

## Introduction

Therapeutic monoclonal antibodies (mAbs) are important therapeutic proteins^1^. Bispecific antibodies (BsAbs) have demonstrated enhanced biological functions in many cases^2–5^. While natural antibodies are a ‘Y’ shape formed by two identical antigen-binding Fab arms connected to Fc domains, BsAbs are engineered to have two different antigen-binding Fab arms. As such, BsAbs may facilitate recruitment of cytotoxic T cells to tumor cells^2^, simultaneously inhibit two signaling pathways^3^, increase specificity for cells that express both antigens^6^, shuttle an antibody across the blood-brain barrier^7^, and neutralize HIV-1^4^. Over the past two decades, structure modification of BsAbs by genetic engineering resulted in a range of recombinant BsAb formats^8^. However, the methods producing BsAbs with high efficiency and without mispairing still need further improvement. Up to now, catumaxomab and blinatumomab are the only two BsAbs approved on the market, partly due to the challenges in producing BsAbs.

BsAbs may present as bearing or lacking an Fc region. The BsAbs without Fc contain only two V_L_ and two V_H_ regions with artificial linkers, such as tandem scFv^2^ and diabodies^9^. These types of molecules cannot bind to the neonatal FcRn receptor, leading to rather rapid renal elimination *in vivo*. For example, the half-life of blinatumomab is only 1.25 ± 0.63 h^10^. In addition, the artificial linker could be potentially immunogenic^11^. The Fc region in a full-length antibody molecule facilitates purification through protein A binding and contributes to its solubility and stability. Additionally, Fc mediates effector functions, such as antibody-dependent cellular cytotoxicity (ADCC) and complement-dependent cytotoxicity (CDC), and extends *in vivo* half-life due to the increased size and FcRn-mediated recycling processes. Therefore, BsAbs with an Fc domain would be more desirable in many therapeutic applications.

Efforts to create bispecific antibodies with an Fc region resulted in dual variable domain IgGs (DVD-Ig)^5^ and IgG-scFv^12^, which are tetravalent unnatural formats, different in size and geometry from conventional IgG antibodies and may create potential immunogenicity^13^. Clinical applications prefer monovalent antigen recognition and natural IgG structure without potentially immunogenic linkers^13, 14^. Catumaxomab, containing complete nonhuman sequences, had immunological responses that accelerate clearance and inhibit its function in humans^15^. The “Dual Acting Fab (DAF)” approach can develop BsAb with human sequences, but is highly dependent on structural properties. It may be impossible to identify an ideal dual specific candidate that exhibits all desired properties^16^.

Chain mispairing is a major problem in making BsAbs. The “knobs-into-holes (KiH)” technology offers a way to minimize the heavy/heavy chains mispairing^17^, but not light/heavy chains mispairing. As the Fab domain is responsible for binding affinity, correct pairing of light/heavy chains is crucial. Solution with “common light chain”^18^ may not be optimal in binding specificity or possible for all BsAbs. A better approach was provided by CrossMab technology^19^. Correct pairing of the light chains is achieved by exchanging the C_H1_ domain of one heavy chain with the C_L_ domain of the corresponding light chain. This approach has been used to create therapeutic BsAbs for anti-virus applications^4, 20, 21^. But in “CrossMab” technology, unnatural domain junctions were generated and natural antibody architecture was replaced. Another approach is to express mAbs separately^22–24^, then combine the two mAbs under mild refolding conditions to form a hybrid BsAb molecule. However, its application is largely limited due to product instability and potential immunogenicity^24^. A similar strategy was presented by Spiess *et al*., who co-cultured two *E*. *coli* strains expressing corresponding half of each mAb that was refolded to synthesize BsAb. Lacking post-translational modification may result in differences in biological functions, stability and *in vivo* half-life^25, 26^. Lewis *et al*. reported an “orthogonal interface mutation” strategy to minimize light/heavy chains mispairing. It requires 15 – 20 mutations that might create immunogenicity^26^. So far, it is only applicable to kappa but not lambda variable domains^26, 27^.

“Protein Trans-splicing (PTS)” has been developed in research laboratories for protein splicing and ligation. In PTS, split inteins are used to create a new peptide bond between their flanking exteins. Split inteins comprise N-terminal fragment (Int^N^) and C-terminal fragment (Int^C^). Fragment complementation leads to reconstitution of the canonical intein fold, recovery of protein splicing activity, and ligation of the exteins^28^. Split inteins have been used for various practical applications, including protein ligation or labeling^29, 30^, protein reconstitution^31, 32^ and protein purification^33^.

We report a platform for synthesizing BsAbs with natural human IgG architecture and elimination of mispaired chains. In this method, two antibody fragments carrying different target-specificities are separately expressed in mammalian cells and subsequently fused to form BsAbs by utilizing the trans-spliced property of the split intein *Npu* DnaE. Three BsAbs were synthesized with this method. The products have been confirmed for their dual binding affinities and expected biochemical-physical properties, as well as associated biological activities.

## Results

### Design of bispecific antibody molecule assembly

**Figure 1:**
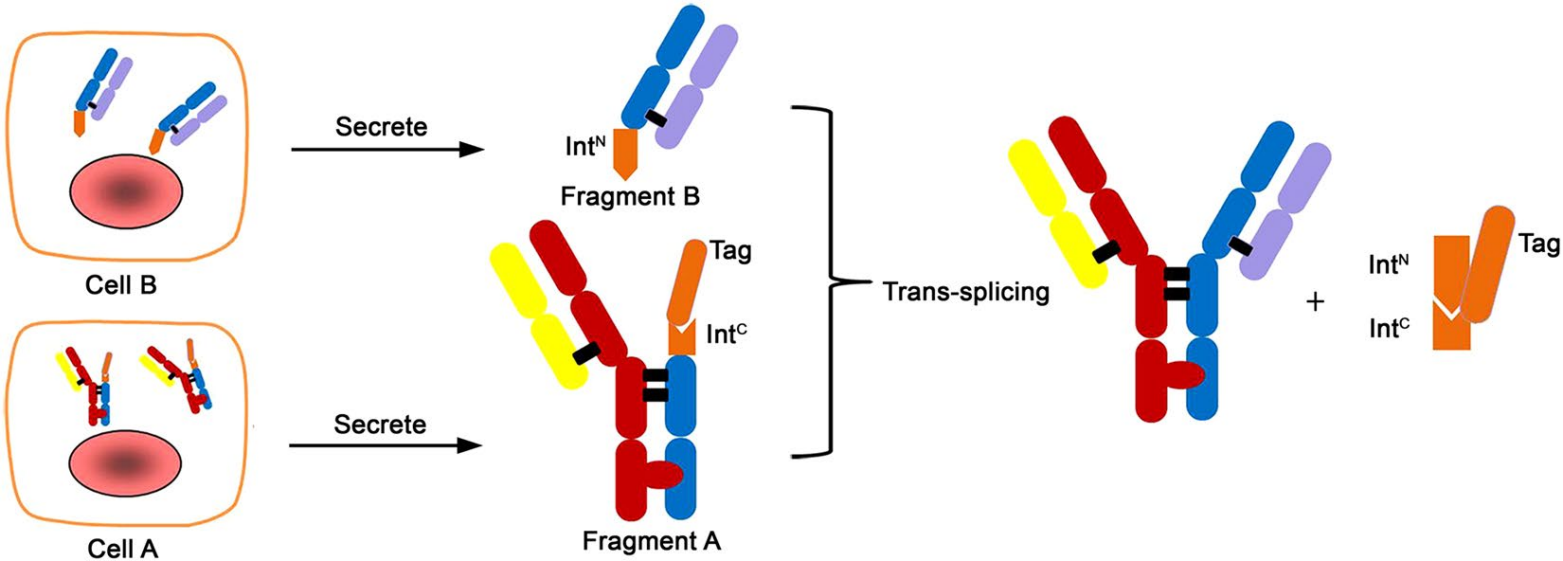
Schematic of the process for bispecific antibody production using the split intein *Npu* DnaE trans-splicing activity. Three steps were involved, the first was the expression of the fragment A and B in mammalian cells respectively; the second was trans-splicing of the fragment A and B; and the last was purification of the BsAb. Fragment A and B were harvested after cell culture separately, then after primary purification followed by trans-splicing reaction, the two fragments were ligated to form the BsAb.

Antibody is composed of two Fab domains and one dimer Fc domain for biological functions. During maturation of an antibody in nature, heavy chains go through dimerization first, followed by light chains pairing with heavy chains^34^. Due to the criticality of antibody molecular assembly, it is preferable to maintain this process, i.e., the pairing of heavy/heavy and light/heavy chains as it occurs naturally. Therefore, we chose to synthesize two antibody arms, one arm with dimer Fc domain and the other without, and both separately expressed in two mammalian cells, so eliminated light/heavy chains mispairing. The hinge region, a region with less functional impact, was selected for conjugating the two arms. The conjugation can be accomplished either chemically or enzymatically. In our method, we chose a split intein for its capability of conjugating two peptides by autocatalytic “PTS”. The two parts of mAbs arms carrying necessary intein components were connected through “PTS” to form a BsAb (Fig. 1). We called this molecular approach “BAPTS” (“Bispecific Antibody by Protein Trans-Splicing”), which solved the light/heavy chain mispairing problem. The “Knobs-into-Holes” or other Fc engineering methods could also be adopted in our molecular construction to enhance correct pairing of the heavy chains. We demonstrated, by characterizing the BsAbs generated from the “BAPTS”, that no differences were expected or detected in antigen-binding behavior of the BsAbs compared to parental mAbs.

### Generation of bispecific antibody by “BAPTS”

**Figure 2:**
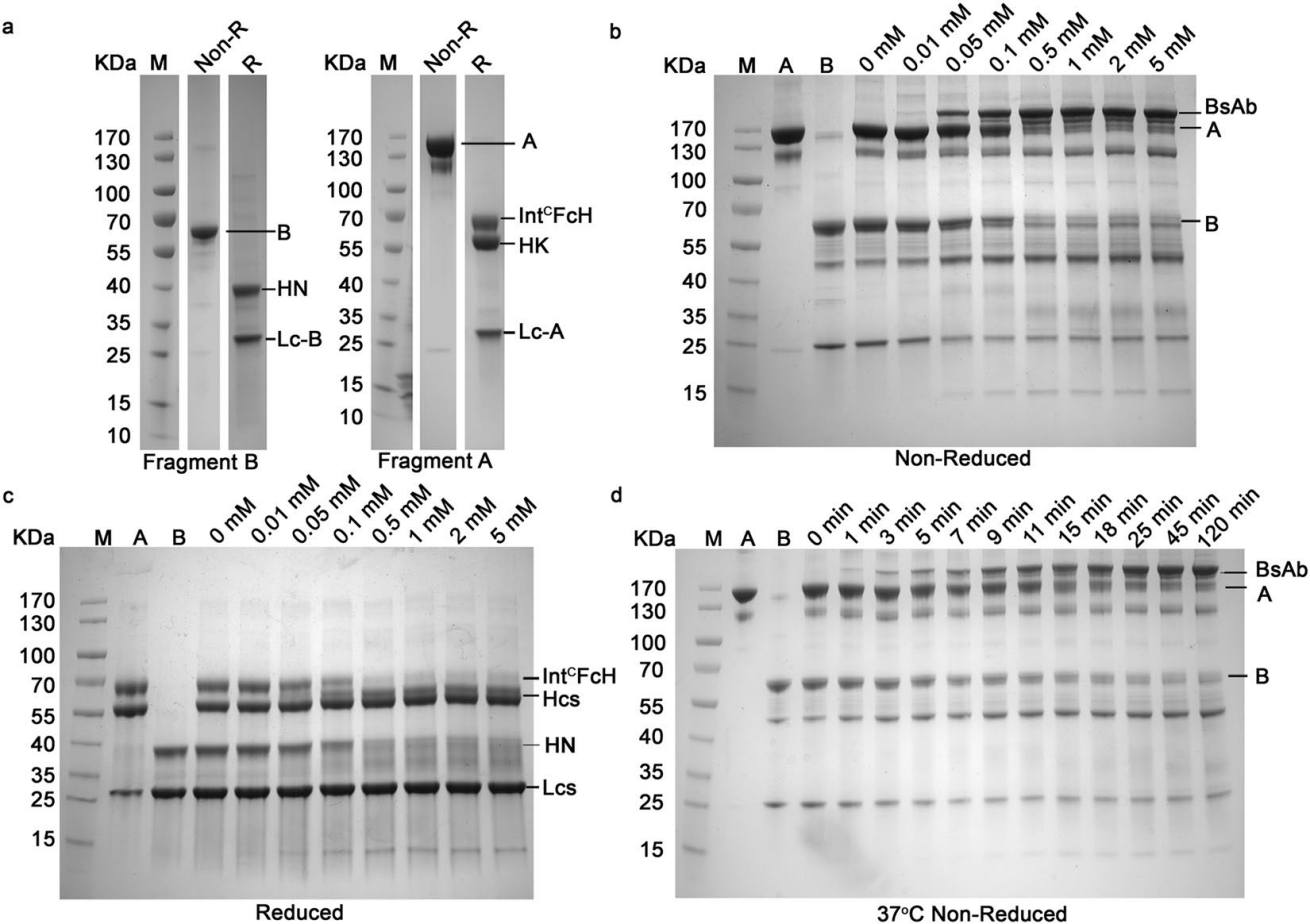
“BAPTS” technology mediated generation of the BsAb (CD3 × HER2). (**a**) SDS-PAGE (4 – 20%) analysis of the fragments A and B under non-reduced and reduced conditions. All lanes were excised from two gels (Supplementary Figure 1a, lanes 4 and 11; Supplementary Figure 1b, lanes 6 and 13) run under the same condition. (**b**,**c**) Catalytic trans-splicing reaction between fragments A and B at 37°C in the absence or presence of various concentrations of reducing agent DTT for 2 hours. The expected trans-spliced product BsAb started to appear at 0.01 mM DTT and the reaction reached plateau at 0.5 mM DTT. (**d**) SDS-PAGE (4 – 20%) of the reaction between fragments A and B performed at 37°C in the presence of 0.5 mM DTT. About 90% of raw material was converted to BsAb within 25 min after the reaction started.

The BsAb fragments A and B (CD3xHER2) were produced by transient expression in HEK293E cells from three expression plasmids (coding CD3-Lc, CD3-HK and Int^C^FcH respectively) for fragment A and two expression plasmids (coding HER2-HN and HER2-Lc) for fragment B (Supplementary Table 1). All antibody fragments were purified via standard protein L affinity chromatography. The fragment A could be reduced into three peptides CD3-Lc, CD3-HK and Int^C^FcH. Likewise, the fragment B could be reduced into two peptides, HER2-HN and HER2-Lc (Fig. 2a). The expression titers for both fragments were in the range between 10–100 mg/L, which is quite normal as for a conventional IgG1 recombinant antibody expressed by transient gene expression system. Fragments A and B were fused by *in vitro* protein trans-splicing reaction. After the reaction, a new band was observed on the SDS-PAGE at the position corresponding in size to the expected splice product BsAb (CD3×ER2). DTT concentration range of 0 – 5 mM was used to facilitate the trans-splicing reaction. The reaction reached the plateau at 0.5 mM DTT, as no further obvious increase of trans-spliced product from 0.5 mM to 5 mM (Fig. 2b,c). A concentration of 0.5 mM DTT did not have an impact on antibody structure. We also observed some other impure bands showing increase or decrease besides of disappearance of reaction substrate fragments A and B (Fig. 2b), Int^C^FcH and HN (Fig. 2c). The band between 40 KDa and 55 KDa represented dimeric light chain and the band at 25 KDa represented the monomeric light chain (Fig. 2b). In the process of reaction, light chain dimer was formed after re-oxidation while the monomer was decreased correspondingly since DTT could reduce exposed cysteine of free monomeric light chain. The band of 15 KDa was formed due to cleaved Int^N^ and it was increased as the process going (Fig. 2b). As shown in time course experiments at 37°C, 90% of raw material was consumed within 25 min after the reaction started (Fig. 2d). Even at low temperature 4°C, the reaction was nearly completed to generate the product within 2 hours, indicating overall low activation energy required for the reaction (data not shown). The low reaction activation energy confirmed that the “BAPTS” approach is feasible for industrial application.

**Figure 3:**
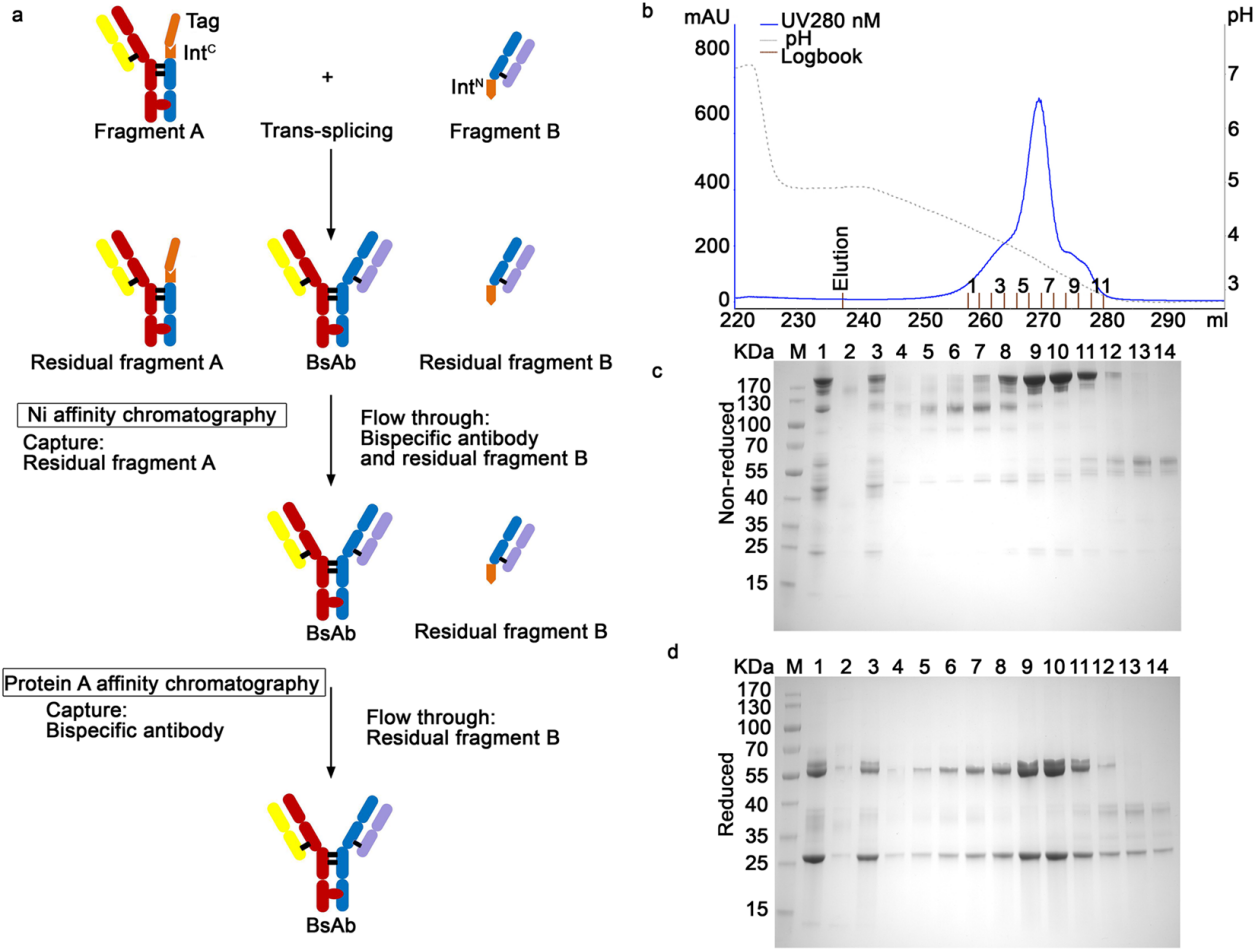
The purification of BsAb (CD3×HER2). (**a**) Purification workflow. In the first step, unreacted fragment A was removed by capturing on Sepharose affinity column through the His-tag. In the second step, the product was isolated from the rest of reaction mixture by protein A affinity chromatography to remove fragment B. (**b**) The chromatography of protein A purification of trans-spliced BsAb (CD3×HER2), Using pH gradient elution from pH 5.0 to pH 2.8. (**c**,**d**) SDS–PAGE (4 – 20%) analysis of the protein A elutions. Lanes: 1, Sample after the trans-splicing reaction of fragments A (CD3) and B (HER2); 2, Ni column Elution; 3, Ni column flow through; 4 to 14, fractions of protein A elution from 1 to 11 respectively.

The product BsAb was purified from the system efficiently through affinity purification steps. Unreacted fragment A was removed by capturing on Sepharose affinity column through the His-tag. The product was isolated from the rest of reaction mixture by protein A affinity chromatography to remove fragment B (Fig. 3). Besides CD3×HER2, two other BsAbs (EGFR×HER2 and CD3×EGFR) were also produced through “BAPTS” with similar outcome, indicating that the “BAPTS” could be used as a generic technology platform for BsAb production.

### Characterization of the bispecific antibodies

**Figure 4:**
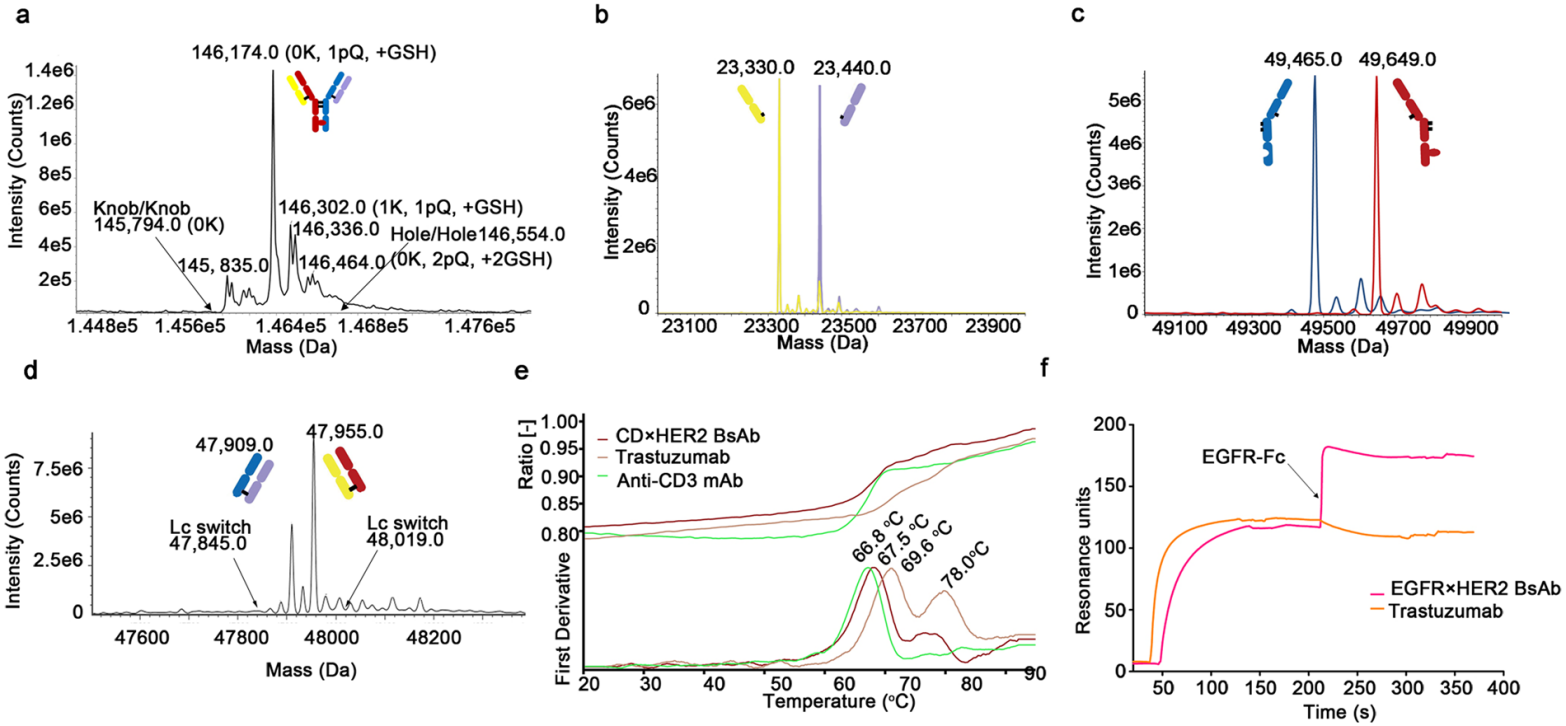
Physico-chemical characterization of BsAbs (post-protein A purification). IMS Q-Tof mass spectrometry, Differential scanning fluorimetry and SPR were performed for BsAb (CD3×HER2). (**a**) Mass spectrometry analysis of the deglycosylated and non-reduced BsAb was performed to demonstrate correct mass for the product without mispairing. Expected mass location for potential knob/knob (K/K), and hole/hole (H/H) homodimers were labeled by the arrows. K, C-terminal lysine presence; pQ, N-terminal pyroglutamic acid cyclization; +GSH, reduced glutathione addition. (**b**,**c**) Mass spectrometry analysis of the deglycosylated and reduced sample to confirm the presence of two different light chains and two different heavy chains. (**d**) Mass spectrometry analysis of the papain digested sample to determine if light chain switching occurred during the BAPTS process. The arrows indicate theoretical masses for the Fab if cognate heavy-light chain pairs had switched. (**e**) Differential scanning fluorimetry measurement of the BsAb (CD3×HER2) and it’s parental mAbs. (**i**) Dual antigen binding behavior of the BsAb (EGFR×HER2) measured by SPR. The BsAb was injected over a surface containing immobilized HER2-Fc antigen followed by injection of EGFR-Fc. Trastuzumab was used as a control.

Purified BsAb (CD3×HER2) was characterized through standard physical and biochemical methods, including mass spectrometry, DSF and SPR. Ion mobility quadrupole time-of-flight (IMS Q-Tof) mass spectrometry was utilized to identify molecular mass and chains assembly of the BsAbs (Fig. 4a–d, Supplementary Figure 2 and Supplementary Table 2). The theoretical molecular mass of the BsAb (CD3×HER2) was confirmed by IMS Q-Tof. The intact antibody was the main peak that exhibited a molecular mass closely matching the predicted mass of the heterodimeric BsAb (CD3×HER2). After deglycosylation, the measured masses of the BsAb (CD3×HER2) were 146,174 Da, 146,302 Da and 146,336 Da, which were agreed with the theoretical molecular masses of the intact molecular C-terminal lysine variants and oxidative modification variants. There was no evidence of knob/knob or hole/hole homodimer formation, indicating that heavy chain mispairing was below limit of detection (Fig. 4a, Supplementary Figure 2a,b and Supplementary Table 2). After deglycosylation and reduction, the BsAb (CD3×HER2) clearly showed the presence of two different light chains (23,330.0 Da and 23,440.0 Da) and two different heavy chains (49,465.0 Da and 49,649.0 Da) (Fig. 4b,c, Supplementary Figure 2c,d and Supplementary Table 2). Sample of Fig. 4a was deglycosylated without reducing, and hole/hole dimer was modified with 2 GSH, leading to higher hole/hole dimer comparing to knob/knob dimer. Sample of Fig. 4c was deglycosylated and reduced, and the MW of knob is higher than hole due to removal of GSH after reduction. Further more, the BsAb (CD3×HER2) also maintained the correct pairing of the light chains to cognate heavy chains along with trans-splicing reaction, as confirmed by papain digestion (which hydrolyzes Fab domains from Fc) followed by mass spectrometry analysis (Fig. 4d, Supplementary Figure 2e,f and Supplementary Table 2). DSF study was performed to assess the overall stability of the BsAbs. While the two parental IgGs (trastuzumab and anti-CD3 mAb) have different onset temperatures of 60.9°C and 57.6°C and different T_m_ of 69.6°C and 66.8°C, the BsAb (CD3×HER2) exhibited an onset temperature of 58.5°C and T_m_ of 67.5°C (Fig. 4e and Table 1). The affinities (monovalent) for the BsAb (CD3×HER2) and the parental antibodies were determined by SPR to confirm that the affinities of the BsAb to their respective ligands were consistent with those of the parental antibodies (Table 2 and Supplementary Figure 3). In addition, the BsAb (EGFR×HER2) binding to both targets were demonstrated by a sandwich SPR assay (Fig. 4f). The results of characterization are consistent with our design expectations, i.e., the BsAbs stably maintained target binding affinities, likely due to the natural maturation of Fab. Our results also indicated that “BAPTS” is an efficient approach to overcome chain mispairing problems.

**Table 1:** Onset temperature and Tm of the BsAb (CD3×HER2) and it’s parental mAbs.

Antibody	Onset	Tm
CD3×HER2 BsAb	58.5°C	67.5°C
Trastuzumab	60.9°C	69.6°C
Anti-CD3 mAb	57.6°C	66.8°C

**Table 2:** Comparison of the affinity of the BsAb (CD3×HER2) and it’s parental mAbs.

Antibody	K_a_ (1/Ms)	K_d_ (1/s)	K_D_ (nM)
CD3×HER2 BsAb (CD3)	7.723E+4	1.202E-3	15.56
Monovalent anti-CD3 mAb (CD3)	1.455E+5	3.331E-3	22.81
CD3×HER2 BsAb (HER2)	4.894E+5	5.976E-5	0.1221
Monovalent Trastuzumab (HER2)	4.466E+5	9.046E-5	0.2026

### *In vitro* biological activity

**Figure 5:**
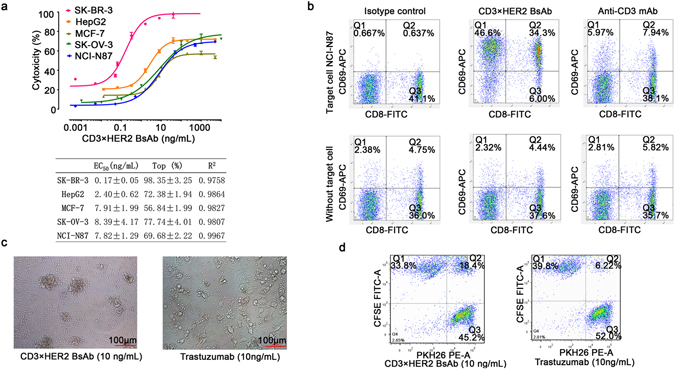
*In vitro* bioactivity of BsAb (CD3×HER2). (**a**) Target-dependent T cell mediated cytotoxicity of BsAb (CD3×HER2) was detected using LDH release assay. Effecters human PBMCs, E:T ratio 10:1, time point of 20 hours. The EC50 values were calculated by fitting the dose-response curve with Graphpad Prism software. R^2^ represents the square of correlation coefficient. (**b**) T cell activation was detected by staining cells for CD8 and CD69 followed by FACS analysis. Effectors CD3^+^ T cells, target NCI-N87 cell line, E:T ratio 10:1. The upper panel showed the stimulation of human T cells by 100 ng/mL BsAb (CD3×HER2) or anti-CD3 mAb in the presence of target cells, while the bottom panel was lack of target cells. (**c**) Photographs of the redirection of T cells to cancer cells by 10 ng/mL BsAb (CD3×HER2). Effecters human PBMCs, target SK-BR-3 cell line, E:T ratio 10:1, time point of 20 hours. (**d**) FACS analysis of the redirection of CD3^+^ cells to cancer cells by BsAb (CD3×HER2). Jurkat (CD3^+^) cells were labeled by PKH26 (PE-A) and NCI-N87 cells were labeled by CFSE (FITC-A) separately. Then the two cells were mixed at equal ratio and treated with 10 ng/mL BsAb (CD3×HER2) or trastuzumab for 30 minutes. Data points in the figure represent the mean of three samples; error bar, SEM.

One panel of cancer cell lines with predetermined numbers of HER2 and EGFR molecules on the cell surface membrane were selected for cytotoxicity studies of the BsAb (CD3×HER2) (Supplementary Figure 4a). The bispecific antibody can induce tumor cell lysis. Typically the T-cell-receptor–associated CD3ε component on T cells is engaged with one arm of the BsAb, whereas the other arm engages with a tumor-specific HER2 antigen. HER2 amplified cell lines were significantly more sensitive to the BsAb (CD3×HER2) than those with low levels of HER2. Notably, cancer cell lines such as McF-7 and HepG2 appeared insensitive to trastuzumab were sensitive to BsAb (CD3×HER2). Additionally, signs of T-cell activation (expressing CD69 and secreting IL-2) were detectable after the BsAb (CD3×HER2) treatment (Fig. 5b and Supplementary Figure 4b,c). Only in the presence of target cells, both CD4^+^ and CD8^+^ T cells could be fully activated by BsAb (CD3×HER2), while slightly activated by anti-CD3 mAb. In addition, after the treatment of BsAb (CD3×HER2), T cells were apparently redirected to tumor cells (Fig. 5c). FACS analysis results also showed a higher ratio of CFSE^+^/PKH26^+^ cells after the treatment of BsAb than trastuzumab, which indicated that BsAb could efficiently bind CD3^+^ Jurkat cells and HER2^+^ NCI-N87 cells together (Fig. 5d). “BAPTS” not only maintained BsAbs’ affinity but also appeared to have better *in vitro* bioactivity than the parental mAb.

### *In vivo* biological activity

**Figure 6:**
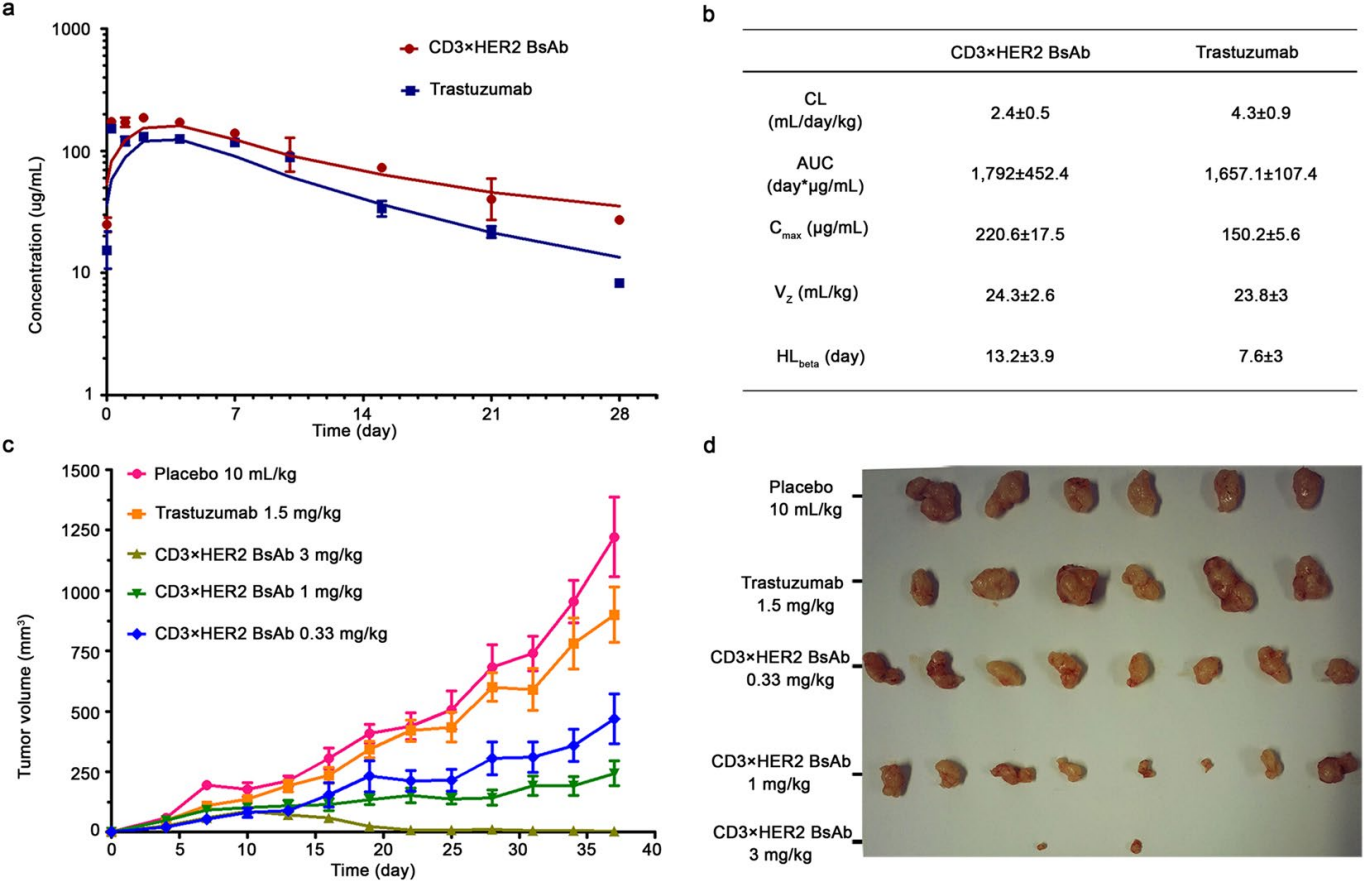
*In vivo* PK profile and tumor growth inhibition bioactivity of the BsAb (CD3×HER2). (**a**) *In vivo* PK analysis of the BsAb and trastuzumab in Balb/c mice (N = 5). Single i.p. doses of 5 mg/kg BsAb or trastuzumab were injected into Balb/c mice. Test animals’ serum samples were assayed by ELISA. (**b**) PK parameters of trastuzumab and the BsAb. Data fitted to the non-compartmental analysis model. (**c**) BsAb inhibited growth of subcutaneously transplanted NCI-N87 cell. NCI-N87 cells were injected together with unstimulated human PBMCs from healthy donors. Mice (N = 8) were treated with 0.33 mg/kg, 1 mg/kg and 3 mg/kg i.p. dose of the BsAb and 1.5 mg/kg dose of Trastuzumab once a week. (**d**) Photographs of excised tumors at the end of the experiment. Error bar, SEM.

*In vivo* pharmacokinetic (PK) parameters of the BsAb (CD3×HER2) and trastuzumab were analyzed in male Balb/c mice. Mice were administered intraperitoneally (i.p.) a single dose of 5 mg/kg. The BsAb (CD3×HER2) displayed a biphasic disposition, which was a typical display of an IgG1 with a short distribution and slow elimination phases. PK parameters were consistent with those of the parental antibodies (Fig. 6a,b). In addition, *in vivo* tumor inhibition by BsAb (CD3×HER2) and trastuzumab was evaluated in female NOD/SCID mice. NCI-N87 cells were grafted together with non-activated human PBMCs from healthy donors to the mice subcutaneously. Mice were dosed intraperitoneally on a weekly schedule with 0.33 mg/kg, 1 mg/kg and 3 mg/kg of BsAb (CD3×HER2) and 1.5 mg/kg of trastuzumab control or negative control PBS 10 mL/kg as placebo, starting on the 1st day after inoculation. As data shown, low dose BsAb (CD3×HER2) had much stronger inhibitory effect on tumor growth compared with trastuzumab (Fig. 6c,d). The BsAb produced by “BAPTS” maintained expected PK the same as parental mAb, indicating that hinge region minor modification caused minimum alteration in metabolic profile. “PTS” introduced stable peptide bond effectively held the integrity of BsAb *in vivo*.

## Discussion

Several methods have been developed to produce BsAbs at industrial scale. However, synthesis of full length BsAbs without misfolding or chain mispairing has not been completely achieved. Compared with mAb, manufacturing BsAb has been suboptimal in not only the production yield but also product quality, mainly caused by chain mispairing. In this report, BsAbs were synthesized efficiently by taking advantage of the protein fusion function of a split intein. With this approach, several BsAbs were synthesized at feasible productivity. The BAPTS is a platform that allows correct assembly of two heavy chains and two light chains, derived from possibly any existing or new antibodies, to form bispecific bivalent IgG antibodies without use of any linkers.

The “BAPTS” is a thermodynamically favored reaction, illustrating feasible application of the approach. In addition, a couple of amino acids including one cysteine residue fused with Int^C^ may facilitate the intein trans-splicing reaction as previously reported^35^. Cysteine residues may serve as an additional modification site for connecting either a bioactive peptide or a peptide toxin that may provide additional biological activities in this platform. Furthermore, additional residues could be inserted to increase the distance and the flexibility between the two arms of the BsAbs, which might be needed in optimizing connections between two cells, such as a tumor cell and a T cell. Optimization of the space between cells might further increase biological activity of the BsAbs, which is under further study.

The applicability of this approach has been confirmed by several combinations of mAb arms, including CD3×HER2, EGFR×HER2, and CD3×EGFR, indicating this approach may be broadly suitable for similar molecules (data not shown). Besides of conjugation in the Fab region using “BAPTS”, this approach could also be used for conjugating either bio-active peptides or peptide toxins/drugs to form BsAb drug conjugates or BsAb immunotoxins.

One manufacturing challenge in producing BsAbs is purification of the final product. Most of reported methods have shown that removal of those product-related impurities is particularly difficult. In “BAPTS” system, a His-tag was fused with starting antibody fragments, so both starting fragments as expressed precursors remaining after processing could be removed in the end of production through highly efficient affinity chromatography. The product BsAb was purified through protein A chromatography step to reach excellent yield and good quality (Fig. 3). Purified products were characterized intensively through physical and biochemical methods, *in vitro* bio-activities (T-cell based anti-tumor profiling and T-cell activation), and *in vivo* animal tumor inhibition experiments. Data consistently suggested that the BsAbs generated through “BAPTS” contain bi-affinity towards either CD3×HER2 or CD3×EGFR or EGFR×HER2 with similar K_D_ as reference molecules^36, 37^. Using a comparable dose as trastuzumab to the NOD/SCID mice, tumors were inhibited significantly more by the BsAb molecule than by trastuzumab, which is consistent to the previous reports^36, 38^. The final product was intensively characterized by utilizing mass spectrometry to confirm mispairing product-related impurities were under limit of detection.

Although the “BAPTS” platform generated three correctly assembled BsAbs, further work is needed to determine whether this approach can be applicable to all IgG types (IgG1, IgG2, IgG3, IgG4, κ light chain and λ light chain) and to demonstrate production at large scale. One can also utilize the system for stocking one mAb arm as a generic partner for a series of products. For example, an anti-CD3 antibody could be a common partner used for conjugating with several other antibody fragments to form a number of BsAbs. This platform technology could become a highly efficient methodology to develop many BsAbs.

In summary, “BAPTS” allowed correct assembly of heavy/heavy chains and light/heavy chains, derived from existing antibodies tested in this report and potentially any other antibodies, to form bispecific bivalent natural architecture IgG antibodies without the use of any synthetic linkers. With the use of this platform, no chain mispairing was observed in the product.

## Methods

### Plasmid construction

All antibody genes and *Npu* DnaE genes were ordered as gene syntheses and cloned via unique restriction sites using standard cloning procedures into separate expression vectors pCEP4 (Invitrogen) enabling secretory expression in HEK293E cells growing in suspension. *Npu* DnaE^N^ was cloned behind the C_H1_ domain of Fab, and CD40 extracellular region fused *Npu* DnaE^C^ was cloned before the Fc domain. A detailed description of the expression plasmids used in this study can be found in the Supplementary Table 1. The construct number 1, 2 and 3 were used to express fragment A, and the construct 4 and 5 were used to express fragment B.

### Transient antibody expression in HEK293E

Each plasmid was prepared using the TM Endo-free Plasmid Maxi Kit (Omega Bio-Tek) following manufacturer’s instructions. Cells were centrifuged prior to transfection and re-suspended at a cell density of 6 million cells/mL in freestyle 293 medium. The DNA was diluted with freestyle 293 medium containing supplements to a concentration of 40 μg/mL and mixed with 25-KD polyethyleneimine (PEI; Polysciences) (DNA:PEI = 1:5, w:w) for an incubation at room temperature for 10 – 15 minutes. Half microgram of plasmid DNA (with an equal ratio of each plasmid) was used in one milliliter of transfection volume. Then the mixture of DNA and PEI was added to the cell culture. Four hours after transfection, cells were diluted with SFM4 HEK293 medium to a cell density of 3 million cells/mL, and valproic acid (VPA, Sigma-Aldrich) was added to a final concentration of 3.8 mM. Twenty four hours post-transfections, 20% TN-1 was added to the culture to a final concentration of 0.5% (w:v). Supernatant either at the time of one week post-transfection or with the cell viability under 50% was taken for analysis or processing^39, 40^.

### Antibody isolation and purification

A simplified expression and purification schematic was developed in Fig. 3a. Briefly, Antibody fragments were purified by protein L affinity chromatography (GE Healthcare) from filtered cell culture supernatants referring to standard protocols. The supernatants were loaded and equilibrated with binding buffer (20 mM sodium phosphate, 500 mM NaCl, pH 7.4). Elution of antibodies was achieved at pH 2.8 (0.1 M sodium citrate) with a 20 CV linear gradient from 0% to 100%, followed by immediate neutralization of the sample. The samples were dialyzed overnight to splicing buffer (10 mM Tris-HCl, 0.5 M NaCl, pH 7.9), followed by splicing reaction of the antibody fragments catalyzed by the split intein. Imidazole were added into the splicing product at 30 mM final concentration, then the reaction mixture went into the Ni Sepharose affinity chromatography (GE Healthcare) and flowthrough containing the BsAb was collected. The flowthrough collect was further purified through protein A affinity chromatography (GE Healthcare). Antibody was eluted at pH 2.8 (0.1 M sodium citrate) with an elution of 20 CV linear gradient from 0% to 100%. The eluates were neutralized immediately, dialyzed overnight to PBS, and filter-sterilized over 0.22 μM dead-end filters. The protein concentration of the samples was measured by BCA protein assay kit.

### Intein catalyzed antibody splicing

For DnaE mediated protein trans-splicing, 10 μM fragment A was mixed with 10 μM fragment B in splicing buffer (10 mM Tris-HCl, 0.5 M NaCl, pH 7.9). DTT was added to a final concentration of 0, 0.01, 0.05, 0.1, 0.5, 1, 2 and 5 mM. After incubation at 37°C for 2 hours, the splicing reaction was stopped by removing reducing agents (DTT) through dialysis overnight at 4°C.

### Mass spectrometric determination of bispecific antibody assembly

The total mass of deglycosylated and reduced as well as papain digested bispecific antibodies were determined by ion mobility quadrupole time-of-flight (Acquity VION IMS Q-Tof, waters). Briefly, the purified BsAb samples (1.5 mg/mL) were concentrated into 50 mM ammonium bicarbonate by a factor of 10 (15 mg/mL) by filtration through 30 KDa MWCO membranes. The concentrated sample (0.5 – 1 mg) was deglycosylated with 500 U PNGase F (NEB) in 50 mM ammonium bicarbonate, at 37 °C for 24 h at a protein concentration of up to 15 mg/mL. The deglycosylated sample was diluted with 5% acetonitrile in 0.1% formic acid to obtain 0.5 mg/mL, and used for LC/MS analysis (1 μL). Sample was divided into two equal aliquots. The deglycosylated sample was diluted with 50 mM ammonium bicarbonate to obtain 0.5 mg/mL, and then diluted sample was incubated with 20 mM DTT (Sigma) for 60 min at 50°C. The deglycosylated and reduced sample was used for LC/MS analysis (1 μL). The bispecific antibody was partially digested with papain (no cysteine system). For the digestions, stock BsAb was diluted with cysteine free papain digestion buffer (1 mM EDTA, 50 mM sodium phosphate buffer, pH 6.3) to obtain 0.5 mg/mL. Papain was activated by adding one papain suspension (10 mg/mL, sigma) to nine parts freshly prepared activation buffer (1 mM EDTA, 10 mM cysteine, 50 mM sodium phosphate buffer, pH 7.0), and incubating at 37°C for 15 min. The excess cysteine was removed by buffer exchange against 6 volume of cysteine free digestion buffer. The activated papain was then added to the BsAb solution at enzyme: BsAb ratio of 1% (w/w), and incubated at 37°C for 2 h. Followed, the papain digested sample was diluted with 5% acetonitrile in 0.1% formic acid to obtain 0.5 mg/mL, and used for LC/MS analysis (1 μL). All of three sample were subsequently separated by RP-UPLC (Acquity I-Class, waters) on ACQUITY UPLC Protein BEH C4 column (300 Å, 1.7 μm, 2.1 mm × 50 mm, waters); eluents were 0.1% FA in water (eluent A) and 0.1% FA in acetonitrile (eluent B) and the flow rate was 300 μL/min. The column was heated at 80°C to enhance separation. The UPLC was directly coupled with the Waters Acquity VION IMS Q-Tof mass spectrometer. The MS signals were collected and deconvoluted with UNIFI 1.8 software of Waters.

### Affinity measurement of the bispecific antibodies

Affinity of the antibodies (bispecific antibodies and monovalent antibody fragments) was determined using surface plasmon resonance (Biacore T200, GE). The Fragments A and B are monovalent for CD3 and HER2, they are used as monovalent controls. Human HER2-Fc and CD3D×CD3E heterodimer were immobilized to a CM5 chip surface using standard 1-ethyl-3 (3-dimethylaminopropyl) carbodiimide (EDC)/N-hydroxysuccinimide (NHS) amine coupling protocols. The running buffer (and dilution buffer) was PBS + 0.1% Tween 20. The chip surface was regenerated by 0.1 M glycine, pH1.5. The concentration series were fit to a 1:1 binding model to determine the binding (K_a_) and dissociation (K_d_) rate constants and the equilibrium dissociation constant (K_D_). To demonstrate simultaneous binding, human Her2-Fc was coupled to a CM5 sensor chip as described above. The bispecific antibody (EGFR×HER2) was injected for 1 min followed by 1-min injection of EGFR-Fc (at a concentration of 10 μg/mL). Surfaces were regenerated using 10 s injections of 0.1 M glycine, pH 1.5. Trastuzumab was injected as controls.

### Differential scanning fluorimetry analysis

Thermal stability of the product was measured by differential scanning fluorimetry (DSF) using the Prometheus NT. 48 instrument. For this, 10 μL of 200 μg/mL antibody was loaded onto nanoDSF grade standard capillaries. Thermal unfolding of antibody was analyzed in a thermal ramp from 20°C to 95°C with a heating rate of 1°C/min.

### *In vitro* cytotoxicity assays

Target cells (1 × 10^4^ cells/well) were seeded on 96-well cell culture plates (Corning). Second day antibodies were pre-incubated for 30 minutes in 37 °C in culture medium(no phenol RPMI 1640 + 10% FBS + 2 mM L-glutamine) before adding the human PBMCs in a 10:1 E/T ratio. The cells were incubated for an additional 20 hours before detecting death by measuring the lactate dehydrogenase activity from the medium using CytoTox 96® Non-Radioactive Cytotoxicity Assay Kit (LDH; Promega). All measurements were done in triplicate. The percentage of cytotoxicity was calculated as follows: cytotoxicity% = (experimental lysis – spontaneous effector lysis − spontaneous target lysis)/(maximum target lysis − spontaneous target lysis) × 100.

### Analysis of T-cell activation

Early signs of T-cell activation (CD69) were detectable after bispecific antibody treatment was initiated. CD3^+^ positive T cells were isolated by dynabeads (11365D, Invitrogen) according the standard protocol. Purified CD3^+^ T Cells were stimulated by 100 ng/mL BsAbs, mAbs and isotype control, then cultural supernatant and cells were collected. The stimulated CD3^+^ cells were stained with CD8-FITC (BD Biosciences; 555634) and CD69-APC (BD Biosciences; 560967) followed by FACS analysis. Secreted IL-2 in cell cultural supernatant was measure by IL-2 immunoassay kit (R&D Systems; 876970).

### Analysis of T-cell redirection

HER2 positive NCI-N87 cells were labeled with CFSE (Invitrogen; C34554) and CD3 positive Jurkat cells were labeled with PKH26 (Sigma; PKH26GL) according to the manufacturer’s protocols. The labeled cells were mixed at equal ratio, then treated with BsAb (CD3 × HER2) or trastuzumab for 30 minutes at 4°C. FACS analysis was performed to detect CFSE^+^/PKH26^+^ cell assembly.

### Pharmacokinetic study in mice

All animal studies were conducted under Institutional Animal Care and Use Committee of the School of Pharmacy of Shanghai Jiao Tong University. Male Balb/c (6 – 8 weeks old) mice (N = 5/group) were dosed intraperitoneally (i.p.) with either BsAb (CD3×HER2) or trastuzumab at 5 mg/kg. Serum samples were assayed for human IgG by ELISA and PK parameters were determined with a non-compartmental analysis model using WinNonlin.

### Xenograft tumor studies

Dosing and monitoring were performed in accordance with guidelines from the Institutional Animal Care and Use Committee of the School of Pharmacy of Shanghai Jiao Tong University. Female NOD/SCID (6 – 8 weeks, Charles River) mice (N = 8/group) were implanted with NCI-N87 cells and human PBMCs. On day 0, 5 million NCI-N87 cells and 1.67 million inactivated human PBMCs in PBS were inoculated subcutaneously. The first treatment was administered 1 day after inoculation. All treatments were administered once a week by intraperitoneal injection.

### Animal experiment statement

All methods were conducted in accordance with guidelines from the Institutional Animal Care and Use Committee of the School of Pharmacy of Shanghai Jiao Tong University, and all experimental protocols were approved by Institutional Animal Care and Use Committee of the School of Pharmacy of Shanghai Jiao Tong University. Informed consent was obtained from all participants donating blood.

### Data availability statement

The datasets generated during and/or analyzed during the current study are available from the corresponding author on reasonable request.

## Electronic supplementary material

Supplementary Information
