# Supplementary material for: Efficient generation of bispecific IgG antibodies by split intein mediated protein trans-splicing system
Source: Sci Rep. 2017 Aug 21;7:8360. doi: 10.1038/s41598-017-08641-3 (PMC5567192; doi:10.1038/s41598-017-08641-3)
Supplement: Supplementary file 1 — Supplementary Information [file 41598_2017_8641_MOESM1_ESM.pdf]

Efficient generation of bispecific IgG antibodies by split intein mediated protein trans-splicing system

Lei Han, Junsheng Chen, Kai Ding, Huifang Zong, Yueqing Xie, Hua Jiang, Baohong Zhang, Huili Lu, Weihan Yin, John Gilly, and Jianwei Zhu

Supplementary Information

Supplementary methods

Cell lines and culture condition. 293C18 Human Embryonic Kidney cells (HEK293E), (CRL-10852, American Type Culture Collection, Manasas, VA) were cultured in a growth medium consisting of a 50/50 mix of the FreeStyle 293 Expression Medium (freestyle 293 medium, Gibco) and the SFM4 HEK293 medium (Hyclone) containing 100 μg/mL of G418 (Invitrogen). The HT-29 cell (ATCC, HTB-38), SK-BR-3 cell (ATCC, HTB-30) and SK-OV-3 cell (ATCC, HTB-77) were grown in McCoy's 5a medium. NCI-N87 cell (ATCC, CRL-5822) was grown in RPMI 1640 medium. U-87 (ATCC, HTB-14), HepG2 cell (ATCC, HB-8065) and McF-7 cell (ATCC, HTB-22) were cultured in DMEM medium, only McF-7 growth medium containing 0.01 mg/mL human recombinant insulin. Ten percent of FBS and 2 mM L-glutamine were added into all media except freestyle 293 medium (Gibco) and SFM4 HEK293 (Hyclone). All cell lines were maintained at 37°C in a 5% (v/v) CO_2 humidified incubator. All cell lines listed were tested negative of mycoplasma.

Isolation of primary lymphocytes. Peripheral blood mononuclear cells (PBMCs) were isolated from blood of healthy donors by density gradient centrifugation using Ficoll-Paque Premium (GE), according to the manufacturer's instructions. Briefly, blood was diluted 1:1 in PBS and layered on top of 15 mL Ficoll in 50 mL tubes. Tubes were subsequently centrifuged at 400×g, at 20°C for 30 min, and PBMCs were

recovered from the plasma–medium interface. Then collected PBMCs were washed one time with PBS and three times with culture medium (no phenol RPMI 1640 + 10% FBS + 2 mM L-glutamine) until supernatant was clear. Isolated PBMCs were re-suspended in culture medium to a final concentration of 7×10^{6} cells/mL.

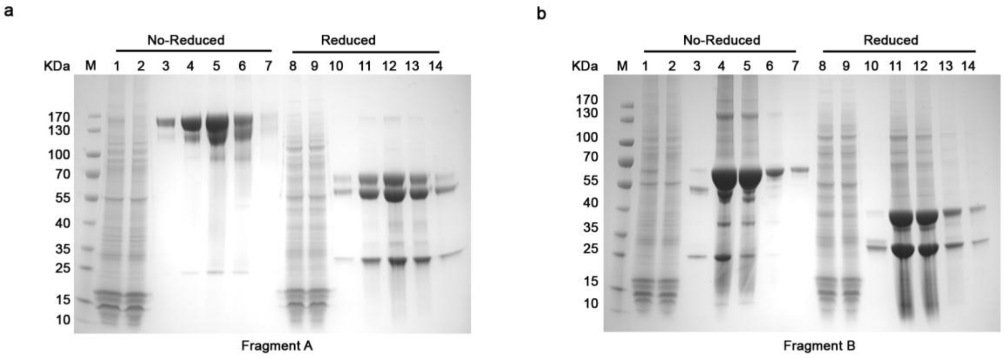

Supplementary Figure 1. The purification of fragments A and B by protein L chromatography. (**a**) SDS-PAGE analysis of the fragment A under non-reduced and reduced conditions. (**b**) SDS-PAGE analysis of the fragment B under non-reduced and reduced conditions. Lanes: 1, cell culture supernatant; 2, protein L flow through; 3 to 7, different fractions of protein L elution. Lanes 8 to 14 are the corresponding samples of lanes 1 to 7 analyzed under reduced condition.

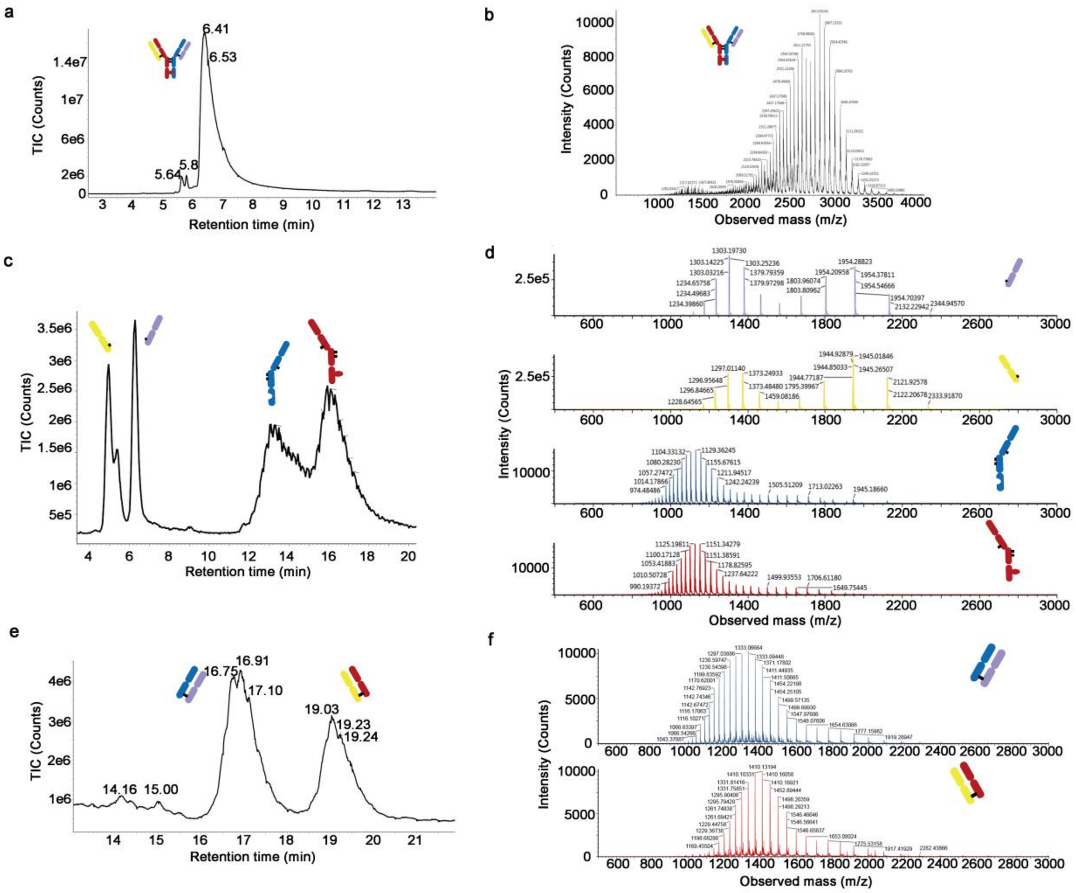

Supplementary Figure 2. IMS Q-Tof analysis of intact BsAb (CD3×HER2), reduced BsAb and Fab domains of BsAb after deglycosylation. (**a**) TIC of the intact BsAb sample. (**b**) The full m/z spectrum with an envelope for the intact BsAb sample which was typical for spectra of antibodies. (**c**) TIC of the reduced BsAb sample. (**d**) The full m/z spectrum with an envelope for the light chains and heavy chains which were typical for spectra of antibodies. (**e**) TIC of the Fab domain of BsAb. (**f**) The full m/z spectrum with an envelope for the Fab domains which were typical for spectra of antibodies.

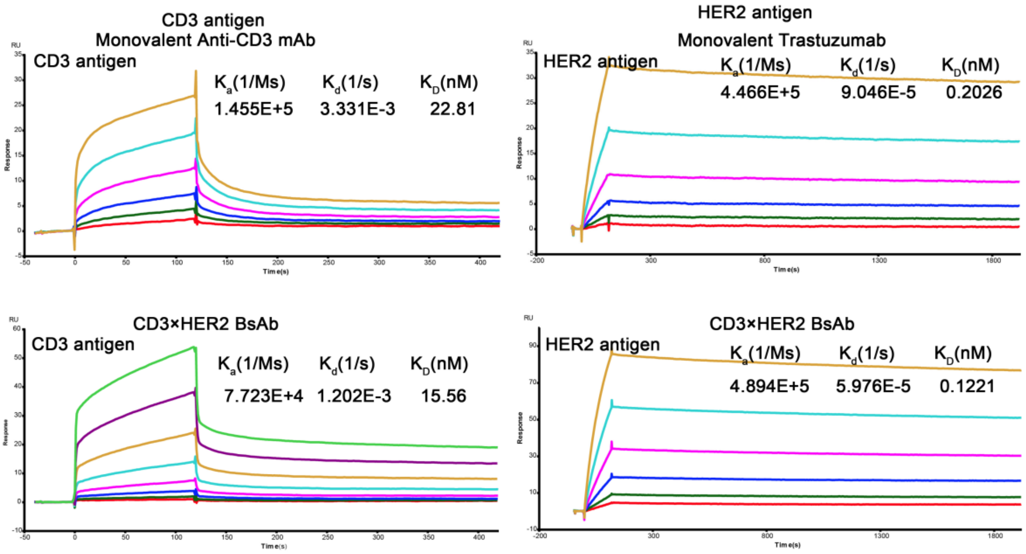

Supplementary Figure 3. Concentration-dependent binding of CD3 and HER2 to the parental antibody and BsAb (CD3×HER2) as determined by surface plasmon resonance. The X axis showed time and the Y axis showed response units. The left panel showed concentration-dependent binding of CD3 to monovalent anti-CD3 mAb and CD3×HER2 BsAb. The right panel showed concentration-dependent binding of HER2 to monovalent Trastuzumab and CD3×HER2 BsAb.

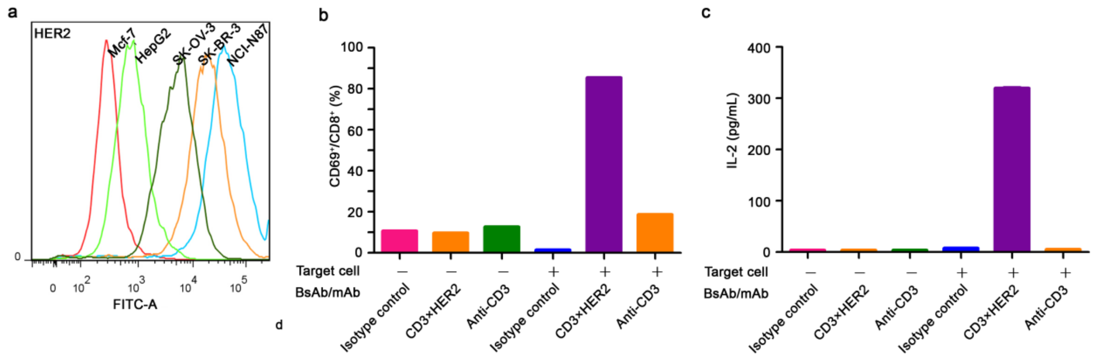

Supplementary Figure 4. HER2 antigen expression level in various cell lines and characterization of T cell activation by BsAb (CD3×HER2) or anti-CD3 mAb. (**a**) HER2 expression levels in cell lines were detected by FACS. (**b**) The expression level of CD69 in $CD8^+$ T cells after the T cell activation (described in Figure 5b). (**d**) The secretion level of IL-2 after the T cell activation. Effectors $CD3^+$ T cells, target NCI-N87 cell line, E:T ratio 10:1, time point of 20 hours.

Supplementary Table 1. Protein numbering, abbreviations and protein sequences, as well as molecular weights, of the proteins used in this study .

Construct	Abbreviation	Protein sequence	Molecular weight (KDa)
1	CD3-Lc	signal sequence + Anti-CD3 mAb-Lc	23.3
2	CD3-HK	signal sequence + Anti-CD3 mAb-Hc (Knob)	49.8
3	Int^{C}FcH	signal sequence + His + CD40ECD + Int^{C} + Fc (Hole)	49.9
4	HER2-HN	signal sequence + Tastuzumab V_{H} + C_{H1} + Int^{N}	35.9
5	HER2-Lc	signal sequence + Tastuzumab-Lc	23.4

Supplementary Table 2. Selected mass intensity data for the BsAb (CD3×HER2).

	Mass (Da)	Intensity (Counts)	Intensity (%)
Intact	145,794	27,803	1.78
	145,835	257,149	16.45
	145,996	186,276	11.92
	146,174	1,563,228	100
	146,302	642,524	41.1
	146,336	555,440	35.53
	146,464	300,489	19.22
	146,554	146,209	9.35
Lc	23,330	6,883,707	72.76
	23,383	623,739	6.59
	23,440	9,460,971	100
	23,493	1,186,686	12.54
	23,601	383,440	4.05
Hc	49,413	161,671	2.59
	49,465	5,669,433	90.88
	49,594	1,429,374	22.91
	49,649	6,238,474	100
	49,776	855,939	13.72
	49,813	475,590	7.62
	49,935	262,325	4.2
Fab	47,685	298,797	3.27
	47,845	189,938	2.08
	47,888	656,962	7.2
	47,909	4,640,387	50.84
	47,955	9,128,259	100
	47,979	954,339	10.45
	48,019	257,002	2.82
	48,116	920,387	10.08
	48,172	870,810	9.54
